# Archaea with a sweet tooth: isolation of novel sugar-degrading thermoacidophiles

**DOI:** 10.1128/msystems.01329-25

**Published:** 2026-02-05

**Authors:** Anthony Kohtz

**Affiliations:** 1 Department of Microbiology, Monash University 214149, Clayton, Victoria, Australia; 2 ARC Research Hub for Carbon Utilisation and Recycling, Clayton, Victoria, Australia; University of Guelph, Guelph, Canada

**Keywords:** archaea, Marsarchaeota, *Tardisphaeria*, polysaccharides, extremophiles, acidophiles, thermophiles

## Abstract

While numerous deep-branching lineages of Archaea have been found over the last two decades, most of them still remain enigmatic and uncultivated. In their article, Prokofeva et al. report the isolation of two species from a thermoacidophilic lineage previously reported as “*Candidatus* Marsarchaeota” and propose a renaming to *Tardisphaeria* (M. I. Prokofeva, A. I. Karaseva, A. S. Tulenkov, A. A. Klyukina, et al., mSystems 10:e00710-25, 2025, https://doi.org/10.1128/msystems.00710-25). Cultivation coupled with genome analysis revealed a strong enrichment and co-occurrence of polysaccharide and sugar metabolisms in these archaea relative to other thermoacidophiles. These polyextreme archaea were also found to make up large abundances (up to 40% relative abundance) in acidic hot springs, indicating they are important for carbon cycling in these environments. These organisms may also host biotechnologically relevant genes for using polysaccharides that are stable at high temperatures and low pH. Overall, these new isolates improve our understanding of archaeal diversity and metabolism and open the door for more studies on these previously inaccessible organisms.

## COMMENTARY

Archaea are a branch of life distinct from Bacteria and Eukaryotes, with wide-ranging implications for understanding our ancestry, metabolic diversity, and biogeochemical cycling on Earth (1–3). While it was long thought that Archaea displayed limited diversity (only two phyla) and were mostly limited to extreme environments, our understanding of Archaea has flourished, with estimates of 20 phyla (comprising some 17,000 genomes) in the most recent release of the Genome Taxonomy Database (r226) (3, 4). Archaea have now been found in extreme and mesophilic environments worldwide, yet the relatively low numbers of archaeal cultures limit our understanding and obscure their biotechnological potential and impacts on biogeochemical cycles (5, 6).

While metagenomically assembled genomes offer abundant information about the potential of organisms, bringing previously uncultured organisms into the lab remains a key approach for understanding their physiology (7–12). Geothermal environments have long been recognized as hot spots of archaeal diversity, with many of the first descriptions of new archaeal lineages stemming from samples collected from these environments (6, 13, 14). Additionally, their extreme conditions lead to reduced complexity of the microbial communities found there, making them tractable for ecological, biogeochemical, and cultivation approaches (15–17).

*Candidatus* Marsarchaeota, a deep branching archaeal lineage, was first described from iron-rich hot springs in Yellowstone National Park (18). Enrichment cultivation, transcriptomics, and genomic analyses showed these archaea are likely aerobic chemoorganotrophs, using oxygen or Fe(III) oxides as electron acceptors (18). Now, from geothermal samples (solfatara and mud spring) in Russia, Prokofeva et al. report the first isolations of two species of *Ca*. Marsarchaeota (19). These isolates were found to be a separate family within *Ca*. Marsarchaeota, and they propose renaming to class *Tardisphaeria*, comprising two families, *Tardisphaeraceae* and *Martarchaeaceae*.

Following the isolation, characterization of the cultures and comparative genomics revealed notable differences in the metabolisms of *Tardisphaeraceae* and *Martarchaeaceae*. While some archaea are specialists, dining sometimes only on a single substrate, other archaea are adapted to a more generalist lifestyle, with a buffet-style menu of substrates they can metabolize. The latter is exemplified by the *Tardisphaeraceae*, which were shown to grow on a wide range of polysaccharides, sugars, and alcohols. Consistent with this, the genomes of these organisms showed enrichment of carbohydrate-active enzymes (CAZymes) and pathways for sugar catabolism. In particular, these new isolates were found to encode the largest set of sugar degradation pathways compared to previously isolated thermoacidophilic archaea and bacteria. In contrast, the previously reported *Martarchaeceae* were found to have a more reduced set of CAZymes, reflecting the differences in the lifestyles of these families.

*Tardisphaerales* were found to be globally distributed across acidic hot springs, acid mine drainage, and rejected coal pile sediments. These environments spanned a pH of 1.5–5.5 and temperatures of 30–85°C, reflecting their establishment in hot acidic habitats. When looking at acidic hot springs in Russia with qPCR and metagenomic sequencing, *Tardisphaerales* were found to make up substantial proportions of the communities (up to 40.7%), suggesting they play key roles in carbon cycling within these habitats. While cultured anaerobically, the *Tardisphaeraceae* strains were found to have tolerance to prolonged oxygen exposure, encoded genes for reactive oxygen species detoxification, and acid tolerance. These traits, when combined with their metabolic diversity, likely underpin their success in polyextreme, hot acid environments.

Open questions involving archaeal metabolism, potential biotechnological applications, and archaeal cell biology will be key areas for future research on *Tardisphaerales*. While they were found to encode the widest range of sugar metabolizing pathways among thermoacidophiles, it remains to be seen whether these are used simultaneously. For biotechnology, these strains may serve as a source of CAZymes that are stable under high temperature and low pH conditions. Key targets were identified, yet their efficacy remains to be tested. For cell biology, abundant and conspicuous intracellular nanocompartments (35–40 nm in diameter) were observed in electron microscopy images of the *Tardisphaerales* isolates. The identity, structure, and functional role of these nanocompartments remain unknown, though they may play important roles in the cell biology of these organisms.

Clearly, more diverse isolates and enrichment cultures of archaea are needed to better represent the wealth of diversity found in sequencing data from environmental samples (6, 20). Overall, the isolation of *Tardisphaeraceae* represents a major step toward bridging the gaps between environmental genomic data and laboratory characterization. As more archaeal cultures become available, the physiological and evolutionary diversity of this domain will continue to expand, reshaping our view of microbial life as a whole and its potential applications.
